# Structural Characterization and Anti-Inflammatory Effects in LPS-Stimulated RAW264.7 Macrophages of an Inulin-Type Fructan (AVP-1-2) Isolated from *Allium victorialis* L.

**DOI:** 10.3390/plants15172687

**Published:** 2026-09-01

**Authors:** Xinyang Guo, Xiaoxiao Xiong, Xueqi Wang, Zeran Wang, Jingran Zhao, Ziyu Wang, Sijia Jiang, Ying Chang

**Affiliations:** 1 College of Life Sciences, Northeast Agricultural University, Harbin 150030, China; 2 Heilongjiang Forest Botanical Garden, Harbin 150040, China

**Keywords:** *Allium victorialis* L., inulin-type fructan, structural properties, anti-inflammatory activity

## Abstract

*Allium victorialis* L. is a significant plant traditionally used as both food and medicine in Northeast China. However, the structural and functional studies of its polysaccharide components remain limited. In the present study, we investigated the anti-inflammatory potential of AVP-1-2 (DP 27), an inulin-type fructan isolated from *A. victorialis*, using an LPS-induced RAW264.7 macrophage model. Its structural characterization was determined through methylation analysis and nuclear magnetic resonance spectroscopy. AVP-1-2 is an inulin-type fructan composed of fructose and glucose. The backbone consists mainly of →1)-β-D-Fruf-(2→, with minor glucose residues and limited branching. The qPCR and ELISA results indicated that AVP-1-2 effectively suppressed the mRNA expression and protein secretion of pro-inflammatory cytokines (TNF-α, IL-6, and IL-1β) in a cellular model. Overall, this work provides new structural and functional insights into *A. victorialis* polysaccharides and offers a theoretical foundation for the comprehensive utilization of *A. victorialis* resources.

## 1. Introduction

Inflammation-related diseases are among the most prevalent health issues globally [1]. The persistent and dysregulated inflammatory response is recognized as a fundamental pathological mechanism underlying various chronic conditions [2]. An overactive inflammatory response is generally characterized by abnormal macrophage activation and the continuous release of pro-inflammatory mediators [3]. Despite notable advancements in the treatment of inflammatory diseases, the associated health and economic burdens remain substantial [1]. Traditional nonsteroidal anti-inflammatory drugs (NSAIDs) frequently lead to organ damage and other adverse effects during treatment [4]. In this context, the pursuit of highly effective and safe anti-inflammatory agents has emerged as a promising therapeutic strategy [5].

Natural polysaccharides from botanical sources are considered promising anti-inflammatory agents owing to their multi-target effects and low toxicity [6]. The anti-inflammatory activity of polysaccharides is closely linked to their fine structural characteristics, including molecular weight, monosaccharide composition, side-chain structure, and spatial conformation [7]. However, the inherent complexity and heterogeneity of natural polysaccharides have hindered a comprehensive understanding of their precise structure–activity relationships [8]. Consequently, the isolation of structurally defined homogeneous polysaccharides and the elucidation of their structure–activity relationships have become a critical focus in current polysaccharide research [9].

The *Allium* genus is one of the oldest cultivated plant species globally, and its polysaccharides (APS) have attracted considerable attention due to their unique properties and health-promoting potential [10]. Research on APS has focused on common species (*Allium sativum* L. [11], *Allium macrostemon* Bunge [12], and *Allium cepa* L. [13]), whereas those from other *Allium* species remain largely unexplored. *Allium victorialis* L. is a rare wild plant found primarily in northeastern China and has a long history of applications as a traditional herb and food [14]. Its abundant phytochemicals, such as sulfur-containing compounds, flavonoids, and essential oils, have been widely reported to exhibit antioxidant, antimicrobial, and cardioprotective activities [15,16]. However, current studies on the polysaccharides of *A. victorialis* (AVP) remain limited, and detailed information on their structure and the associated anti-inflammatory potential remains to be systematically elucidated.

To further investigate AVP, an inulin-type fructan (AVP-1-2) was isolated in the present study. Its detailed structure was characterized by chromatographic and spectroscopic methods. The anti-inflammatory activity was assessed through a cellular model. Collectively, these findings contribute to the resource utilization of *A. victorialis* and support the development of functional foods based on AVP.

## 2. Materials and Methods

### 2.1. Materials and Equipment

*Allium victorialis* L. was collected in May 2019 from Suiyang Forestry Bureau, Heilongjiang Province, China (44°07′25.7″ N, 130°34′04.7″ E) at the vegetative growth stage (Figure S1). The plant material was authenticated by Yuqian Zhou (Professorate Senior Engineer), and stored at Northeast Agricultural University for subsequent analysis. Ethanol (95%, *v*/*v*), n-butanol, dimethyl sulfoxide (DMSO), and papain were supplied from Sigma (St. Louis, MO, USA). Chloroform and phenol were obtained from Sinopharm (Shanghai, China); while trifluoroacetic acid (TFA) was purchased from CNW Technologies (Düsseldorf, Germany). All reagents used in this study were of analytical grade. Monosaccharide standards were purchased from Sigma (St. Louis, MO, USA). DEAE-52 and Sephadex G-200 type column packing materials were purchased from Kalang (Shanghai, China). Chromogenic LAL Endotoxin Assay Kit was obtained from Beyotime Biotechnology (Shanghai, China).

DMEM medium and RAW264.7 cells were purchased from Procell Life Science & Technology (Wuhan, China). Lipopolysaccharide (LPS) was obtained from Yuanye (Shanghai, China). cDNA synthesis kits and Cell Counting Kit-8 (CCK-8) were supplied by Summer Bio (Beijing, China) and Coolaber (Beijing, China), respectively. Beyotime Biotechnology (Shanghai, China) provided the ELISA kits, nitric oxide (NO) detection kits.

The major instruments used in this study included: Industrial rotary evaporator (Hei-VAP, Heidolph Instruments, Schwabach, Germany); Real-time PCR system (Bori Technology, Hangzhou, China); Nuclear magnetic resonance spectrometer (AVANCE III 600 MHz, Bruker, Rheinstetten, Germany); Fourier transform infrared spectrometer (Nicolet iZ-10, Thermo, Waltham, MA, USA); Scanning electron microscope (SEM; SU8010, Hitachi, Tokyo, Japan); Gel chromatography spectrometer (U3000, Thermo, Waltham, MA, USA); Gas chromatography–Mass spectrometry (6890A-5977B, Agilent Technologies, Santa Clara, CA, USA); Ion chromatography spectrometer (ICS 5000+, Thermo, Waltham, MA, USA).

### 2.2. Extraction and Purification of Polysaccharides

The whole plant of *A. victorialis* was initially air-dried in the shade and ground into powder, followed by defatting with petroleum ether at a material-to-liquid ratio of 1:3 (g/mL) for 24 h to eliminate lipophilic components. After filtration, the obtained residue was air-dried. Subsequently, the defatted material was subjected to hot water extraction under different extraction conditions, including material-to-liquid ratios (1:10, 1:15, and 1:20 g/mL), temperatures (85, 90, and 95 °C), and extraction times (2, 3, and 4 h), to determine the optimal conditions for crude polysaccharide yield. Theextracted solution was concentrated using a rotary evaporator and mixed with ethanol (1:3, *v*/*v*), followed by precipitation at 4 °C for 24 h. The precipitate was collected, dissolved in distilled water and treated with papain (8:1000, g/mL) at 55 °C for 6 h. After enzymatic hydrolysis, residual proteins were eliminated using Sevag reagent (chloroform/n-butanol, 4:1, *v*/*v*). The resulting solution was subjected to dialysis (MWCO 3500 Da), and the retained fraction was freeze-dried to obtain crude polysaccharides. This material was subsequently decolorized on macroporous resin AB-8 to obtain the final product (AVP) [17].

For further purification, AVP was dissolved in deionized water and passed through a 0.45 μm membrane filter before loading onto a DEAE Cellulose-52 ion-exchange column (Whatman, Maidstone, UK; 26 mm × 400 mm). Elution was carried out sequentially with deionized water and NaCl solutions at concentrations of 0.1, 0.2, and 0.3 mol/L, respectively, at a flow rate of 4 mL/min. The collected fractions were monitored for polysaccharide content using the phenol-sulfuric acid method at 490 nm [18]. The main fractions were pooled according to the elution profile to obtain two purified fractions, designated AVP-1 and AVP-2. Following dissolution and filtration as described above, AVP-1 was applied to a Sephadex G-200 gel column (Cytiva, Marlborough, MA, USA; 26 mm × 1000 mm) and eluted with deionized water at 1 mL/min, resulting in two homogeneous fractions (AVP-1-1 and AVP-1-2) [19]. The total carbohydrate content of AVP-1-2 was determined by the phenol–sulfuric acid method and was found to be 93.3%. Endotoxin levels in AVP-1-2 (100, 200, and 400 μg/mL) were determined using the Chromogenic LAL Endotoxin Assay Kit (Beyotime, Shanghai, China) and were below the detection limit (<0.01 EU/mL).

### 2.3. Structural Characterization

#### 2.3.1. Molecular Weight Analysis

The molecular weight of AVP-1-2 was determined using GPC-RI-MALS. The system consisted of a U3000 liquid chromatography pump (Thermo, USA) equipped with an Optilab T-rEX differential refractive index detector and a DAWN HELEOS II multi-angle laser light scattering detector (both from Wyatt Technology, Santa Barbara, CA, USA). Two Ohpak columns (SB-803 HQ and SB-805 HQ, 300 × 8 mm; Shodex, Showa Denko, Tokyo, Japan) were connected in series. The mobile phase was 0.1 M NaNO_3_ containing 0.02% (*w*/*w*) NaN_3_. AVP-1-2 was dissolved in the mobile phase at a concentration of 1 mg/mL and filtered through a 0.45 μm filter before injection. Separation was performed at 45 °C with a flow rate of 0.6 mL/min and an injection volume of 100 μL [20]. A dn/dc value of 0.146 mL/g was used for molecular weight calculation. Data were processed using the Zimm fit method in ASTRA software 8.1, and the molecular weight was determined from the main elution peak where both RI and LS signals were clearly above baseline.

#### 2.3.2. FT-IR Analysis

A dried sample of AVP-1-2 (2 mg) was mixed with an appropriate amount of KBr and pressed into thin slices. This spectrum was scanned and analyzed in the range of 4000–400 cm^−1^ using a Nicolet iZ-10 spectrometer (Thermo Scientific, Wilmington, DE, USA) [21].

#### 2.3.3. SEM Analysis

The AVP-1-2 powder was mounted on a stub and sputter-coated with gold powder. The images were observed by scanning electron microscope (SU8010, Hitachi, Japan) at magnifications of 1000×, 3000×, 5000×, and 10,000×.

#### 2.3.4. Congo Red Test

AVP-1-2 (5 mg/mL) was mixed with an equal volume of 100 μmol/L Congo Red solution (Sinopharm, Shanghai, China). NaOH solutions were subsequently added to adjust final concentrations of 0, 0.1, 0.2, 0.3, 0.4, and 0.5 mol/L. After thorough mixing, the solution was allowed to stand at room temperature (RT) for 10 min. The maximum absorption wavelength (λmax) in the range of 400 to 600 nm was measured using a microplate reader (Thermo Fisher, Waltham, MA, USA) [22].

#### 2.3.5. Monosaccharide Composition Analysis

AVP-1-2 (2 mg) was combined with a 2 M TFA (1 mL) solution and heated at 60 °C for 1 h [23]. The reaction mixture was dried under a nitrogen stream in the vial, and the vial was washed five times with methanol. The acidolysis product of AVP-1-2 was obtained after the addition of deionized water. The results of monosaccharide composition were analyzed using an ion chromatograph (ICS 5000+, Thermo Fisher Scientific, USA) equipped with a pulsed amperometric detector (PAD). The chromatographic column used was a Dionex™ CarboPac™ PA20 (150 × 3.0 mm, 10 μm; Thermo Fisher Scientific, Waltham, MA, USA). Separation was performed at 30 °C with a flow rate of 0.5 mL/min and an injection volume of 5 μL. The mobile phase, gradient elution program and column regeneration procedure are described in Table S1. A standard mixture of a total of 13 monosaccharides (fucose, rhamnose, arabinose, galactose, glucose, xylose, mannose, fructose, ribose, galacturonic acid, glucuronic acid, mannuronic acid, and guluronic acid) was analyzed using the same method [24]. Calibration curves were constructed using mixed monosaccharide standards at the concentrations listed in Table S2. The retention times, slopes, and R^2^ values are summarized in Table S3.

#### 2.3.6. Methylation Analysis

AVP-1-2 (3 mg) was dissolved in 500 μL of DMSO. Subsequently, NaOH (1.0 mg) was added and incubated for 30 min, followed by the addition of iodomethane (50 μL) to perform methylation for 1 h. The methylated polysaccharides were hydrolyzed with 2 M TFA (100 μL) at 60 °C for 1 h and evaporated at 30 °C [25]. The hydrolysate was reduced with 2 M ammonia (50 μL) and 1 M NaBD_4_ (50 μL) at RT for 2.5 h. The reaction was terminated with acetic acid (20 μL) and evaporated under nitrogen. Acetic anhydride (250 μL) was added and reacted at 100 °C for 2.5 h. The final product was extracted with dichloromethane and analyzed by GC-MS (Agilent Technologies, Santa Clara, CA, USA, 6890A-5977B). The chromatographic column used was a BPX70 (30 m × 0.25 mm × 0.25 µm, SGE, Melbourne, Australia). Separation was performed at a flow rate of 1.5 mL/min with an injection volume of 1 μL. The column temperature was initially held at 140 °C for 2 min, then increased to 230 °C at 3 °C/min and held for 3 min. PMAA derivatives were identified by matching their retention times and EI fragmentation patterns against the Complex Carbohydrate Research Center (CCRC) PMAA database and published literature [18].

#### 2.3.7. NMR Analysis

AVP-1-2 (50 mg) was dissolved in 0.5 mL of D_2_O and freeze-dried. This process was repeated twice. The NMR spectra, including ^1^H, ^13^C, COSY, HSQC, HMBC and NOESY, were collected at 25 °C using a Bruker Avance III 600 MHz spectrometer (Bruker BioSpin, Rheinstetten, Germany). The obtained NMR data were processed using MestReNova NMR software (version 16.0.0) [26,27].

### 2.4. In Vitro Anti-Inflammatory Activity

#### 2.4.1. Cell Culture and Cytotoxicity Assay

RAW264.7 cells were cultured in DMEM supplemented with 10% fetal bovine serum (FBS) and 1% penicillin–streptomycin at 37 °C in a humidified incubator with 5% CO_2_ and passaged every other day. Cells were seeded into 96-well plates at 1 × 10^5^ cells/mL and incubated for 24 h to allow cell attachment. The cells were then treated with AVP-1-2 (25–800 μg/mL) for 24 h. After treatment, 10 μL of CCK-8 solution was added to each well and incubated for 2 h. The absorbance was recorded at 450 nm. The experiment was independently repeated three times, and cell viability was calculated using the following formula:

$$\mathrm{Cell}\mathrm{viability}(\%)\;=\;\frac{A_{\mathrm{Treat}}-A_{\mathrm{Blank}}}{A_{\mathrm{Control}}-A_{\mathrm{Blank}}\times 100}$$

#### 2.4.2. NO Release Detection

RAW264.7 cells (1 × 10^6^) were seeded into 6-well plates and cultured for 24 h. Cells were pretreated with AVP-1-2 (100, 200, and 400 μg/mL) for 4 h, followed by stimulation with LPS (1 μg/mL) for an additional 20 h [28]. The supernatants were collected after centrifugation (1000× *g* for 10 min). A total of 50 μL of supernatant was mixed with an equal volume of Griess reagent in a 96-well plate and incubated at RT for 30 min. The absorbance was measured at 540 nm using a microplate reader. The NO concentration in each sample was calculated from the standard curve.

#### 2.4.3. Real-Time Quantitative PCR (RT-PCR)

RAW264.7 cells from each group were collected. Total RNA was extracted using TRIzol (Summer Bio, Beijing, China). cDNA was synthesized from isolated RNA with a reverse transcription kit (Summer Bio, Beijing, China) and then amplified on a Bio-Rad CFX Connect system (Bio-Rad, Hercules, CA, USA). Quantification was performed by the 2^−ΔΔCt^ method. The primers are given in Table S4.

#### 2.4.4. Cytokine Secretion Assay

For the quantification of secreted cytokines, RAW264.7 cells were cultured and stimulated under the conditions described in Section 2.4.2. After centrifugation at 1000× *g* for 10 min, the resulting supernatant was subjected to ELISA using commercial kits (Beyotime, Shanghai, China), following the protocol provided by the supplier. The concentrations of TNF-α, IL-1β, and IL-6 were determined from standard curves. The assay was repeated three times independently.

### 2.5. Data Statistics

All data are expressed as mean ± standard deviation (SD), and all experiments were independently repeated three times. Statistical analysis was conducted using GraphPad Prism 10.0. One-way analysis of variance (ANOVA) followed by Tukey’s multiple comparison test was applied for data analysis, and differences were considered statistically significant at *p* < 0.05. Different letters represent significant differences among groups.

## 3. Results

### 3.1. Extraction, Separation and Purification

#### 3.1.1. Optimization of Extraction

**Figure 1:**
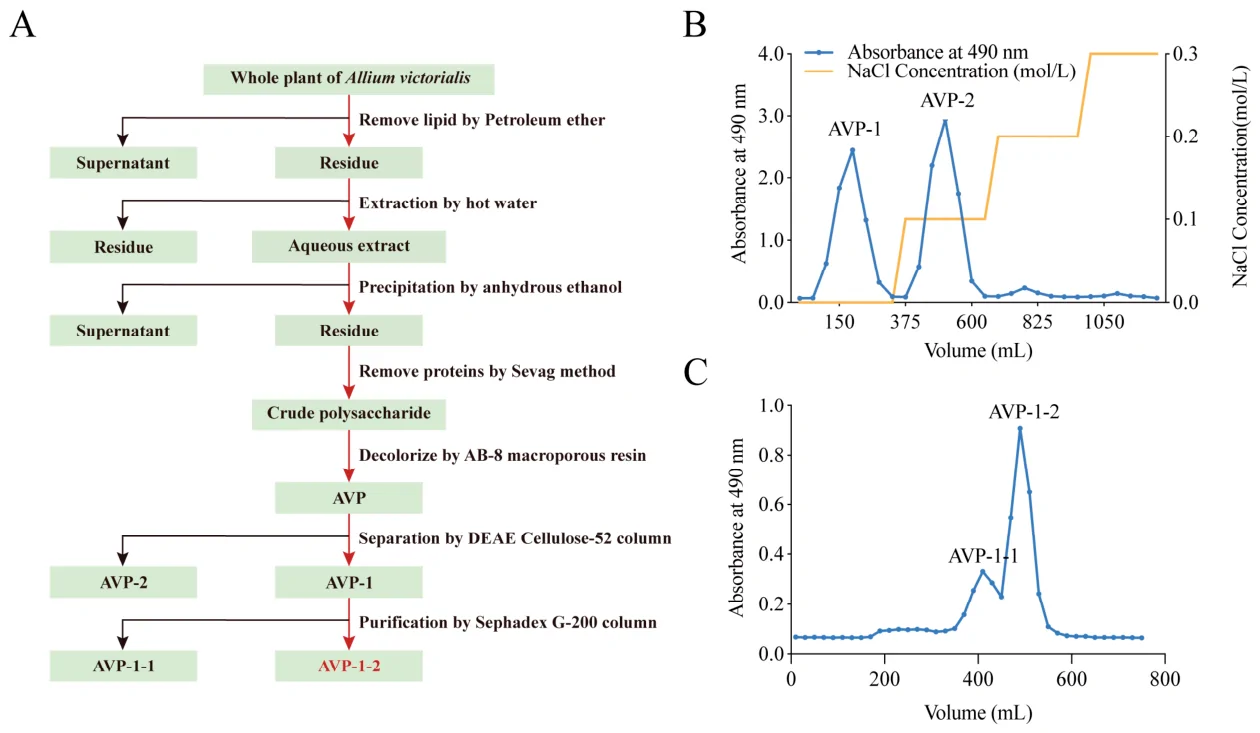
Extraction and purification of AVP-1-2 from *A. victorialis*. (**A**) Flowchart of the extraction and purification procedure; (**B**) DEAE-52 elution curve of AVP; (**C**) Sephadex G-200 elution curve of AVP-1.

The specific procedure of crude polysaccharide from *A. victorialis* is illustrated in Figure 1A. The extraction parameters were optimized using an orthogonal design (Table S5), and the yields obtained under different combinations are presented in Table S6. Range analysis indicated that the factors influencing polysaccharide yield are ranked as follows: A (material–liquid ratio) > B (temperature) > C (time). Within the parameters of the experimental design, the optimal extraction combination identified is a material–liquid ratio of 1:10 (g/mL), an extraction time of 2 h, and an extraction temperature of 85 °C. This method was employed to extract a total of 2 kg of *A. victorialis*, resulting in a yield of 1.632%. The crude polysaccharide was decolorized using macroporous resin AB-8 to obtain AVP.

#### 3.1.2. Separation and Purification

AVP was fractionated by DEAE-52 column using stepwise elution with NaCl (0–0.3 mol/L), yielding two major peaks, AVP-1 (44.5%) and AVP-2 (55.5%) (Figure 1B). AVP-1 was further purified using Sephadex G-200, resulting in two fractions: AVP-1-1 and AVP-1-2, with the latter identified as the predominant component (Figure 1C).

### 3.2. Structural Characterization of AVP-1-2

#### 3.2.1. Monosaccharide Composition of AVP-1-2

**Figure 2:**
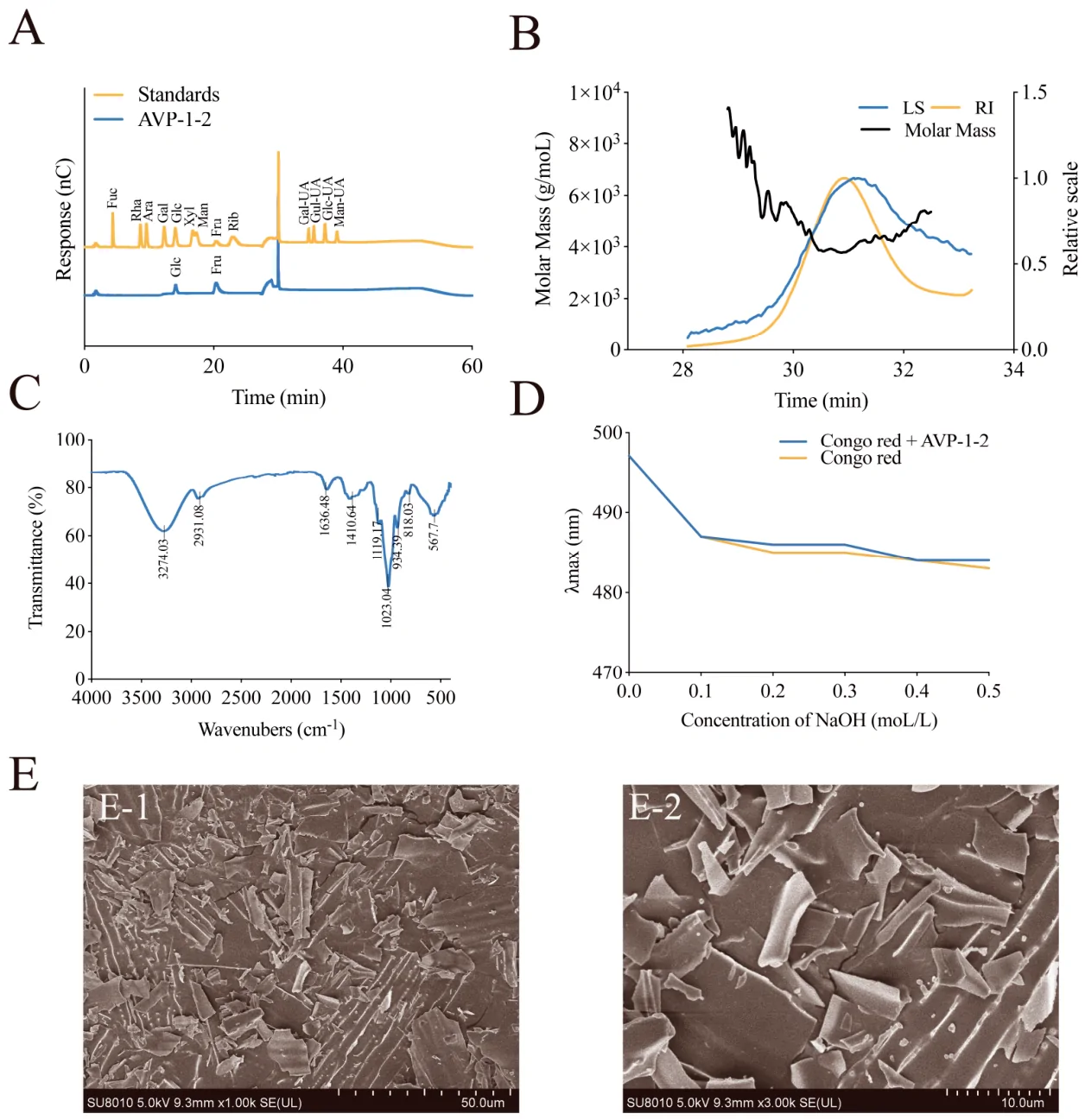
Structural characterization of AVP-1-2. (**A**) Monosaccharide composition of AVP-1-2; (**B**) Absolute molecular weight analysis of AVP-1-2; (**C**) FT-IR spectrum of AVP-1-2; (**D**) Triple helix structure analysis of AVP-1-2; (**E**) Scanning electron microscopy (SEM) images of AVP-1-2: 1000× (**E-1**), 3000× (**E-2**). LS, light scattering detector; RI, refractive index detector.

Monosaccharide composition of AVP-1-2 is illustrated in Figure 2A. Two major monosaccharides (fructose and glucose) were detected as the predominant components of AVP-1-2. No uronic acid components were detected, consistent with the neutral polysaccharide characteristics of AVP-1-2. The detailed analysis of monosaccharide content is summarized in Table 1.

#### 3.2.2. Molecular Weight Analysis of AVP-1-2

The molecular weight distribution of AVP-1-2 is illustrated in Figure 2B. The refractive index (RI) curve exhibited a single major peak with good symmetry, suggesting that AVP-1-2 was mainly distributed within a relatively narrow molecular weight range. The light scattering (LS) curve showed a major peak corresponding to the RI peak, with slight tailing on the LS signal, which may reflect a minor low molecular weight fraction or lower signal-to-noise in this region. This indicated that AVP-1-2 consisted mainly of a relatively homogeneous component, without obvious ultra-large molecular weight components. As shown in Table 2, AVP-1-2 had a molecular weight of 4.403 kDa and a polydispersity index (PDI) of 1.02. The average degree of polymerization (DP) was estimated to be approximately 27 based on the molecular weight and monosaccharide composition. In addition, conformational analysis revealed a slope of approximately 1/3 in the molecular conformation plot, suggesting that AVP-1-2 adopts a spherical shape in solution (Figure S2).

#### 3.2.3. FT-IR Analysis of AVP-1-2

The FT-IR spectroscopy of AVP-1-2 is shown in Figure 2C. The observed absorption peaks at 3274 cm^−1^, 2931 cm^−1^, and 1023 cm^−1^ correspond to the absorption features of polysaccharides. The peak at 3274 cm^−1^ is attributed to the hydroxyl O–H stretching vibration, while the peak at 2931 cm^−1^ is associated with the alkyl C-H stretching vibration of the residual sugar ring [29]. The absorption peak at 1636 cm^−1^ indicates the presence of trace water molecules [22]. The absorption peaks in the region of 1200–1000 cm^−1^ were attributed to C-O-C and C-O stretching vibrations of the pyranose ring and glycosidic bonds [19]. The absorption peaks of AVP-1-2 at 934 cm^−1^ and 818 cm^−1^ suggest the presence of both α and β glycosidic bonds [30]. Furthermore, the absence of a characteristic absorption peak near 1700 cm^−1^ supports the absence of uronic acid in AVP-1-2, consistent with the monosaccharide composition results [31].

#### 3.2.4. Congo Red Assay

We examined the triple helix structure of AVP-1-2 using Congo red analysis (Figure 2D). The λmax of the Congo red group showed a gradual decrease and eventually stabilized as the NaOH concentration increased. Similarly, the λmax of the AVP-1-2 group showed a progressive decrease and then reached a plateau, suggesting that AVP-1-2 lacks a triple-helical conformation [32].

#### 3.2.5. SEM Analysis of AVP-1-2

The surface morphology of AVP-1-2 was examined by SEM (Figure 2E). At magnifications of 1000× (Figure 2E-1) and 3000× (Figure 2E-2), the AVP-1-2 particles predominantly displayed thin lamellar structures interspersed with some squamous formations, lacking prominent depressions and pores. At higher magnifications of 5000× and 10,000×, AVP-1-2 revealed distinct edges, presenting large fragments characterized by sharp contours and relatively smooth surfaces (Figure S3).

#### 3.2.6. Methylation Analysis of AVP-1-2

To elucidate the glycosidic linkages of AVP-1-2, methylation analysis was performed using GC-MS. As shown in Table 3, a total of five linkage types were identified: Fruf-(2→, Glcp-(1→, →1)-Fruf-(2→, →6)-Glcp-(1→, and →1,6)-Fruf-(2→. The mole ratio was determined to be 17.21:1.75:70.48:7.87:2.68. The →1)-Fruf-(2→ derivative was detected as two adjacent peaks at 13.087 and 13.209 min in the total ion chromatogram (Figure S4). This phenomenon may be attributed to the formation of epimeric alditol derivatives during NaBD_4_ reduction of the same fructosyl linkage, leading to two chromatographic peaks. Their EI mass spectra showed the same major diagnostic fragment ions, supporting their assignment as epimeric derivatives of the same linkage (Figure S5). The same explanation applies to the two peaks observed for →1,6)-Fruf-(2→. Fructose-derived residues accounted for over 90% of the detected PMAA derivatives, indicating that the main chain of AVP-1-2 predominantly comprises →1)-Fruf-(2→ residues. Glucose residues were detected only as minor components, mainly as terminal Glcp-(1→ and 6-linked →6)-Glcp-(1→. The molar percentages should be regarded as semi-quantitative and were not used for exact end-group counting or side-chain stoichiometry. These findings preliminarily suggest that AVP-1-2 is an inulin-type fructan [33].

#### 3.2.7. NMR Analysis of AVP-1-2

**Figure 3:**
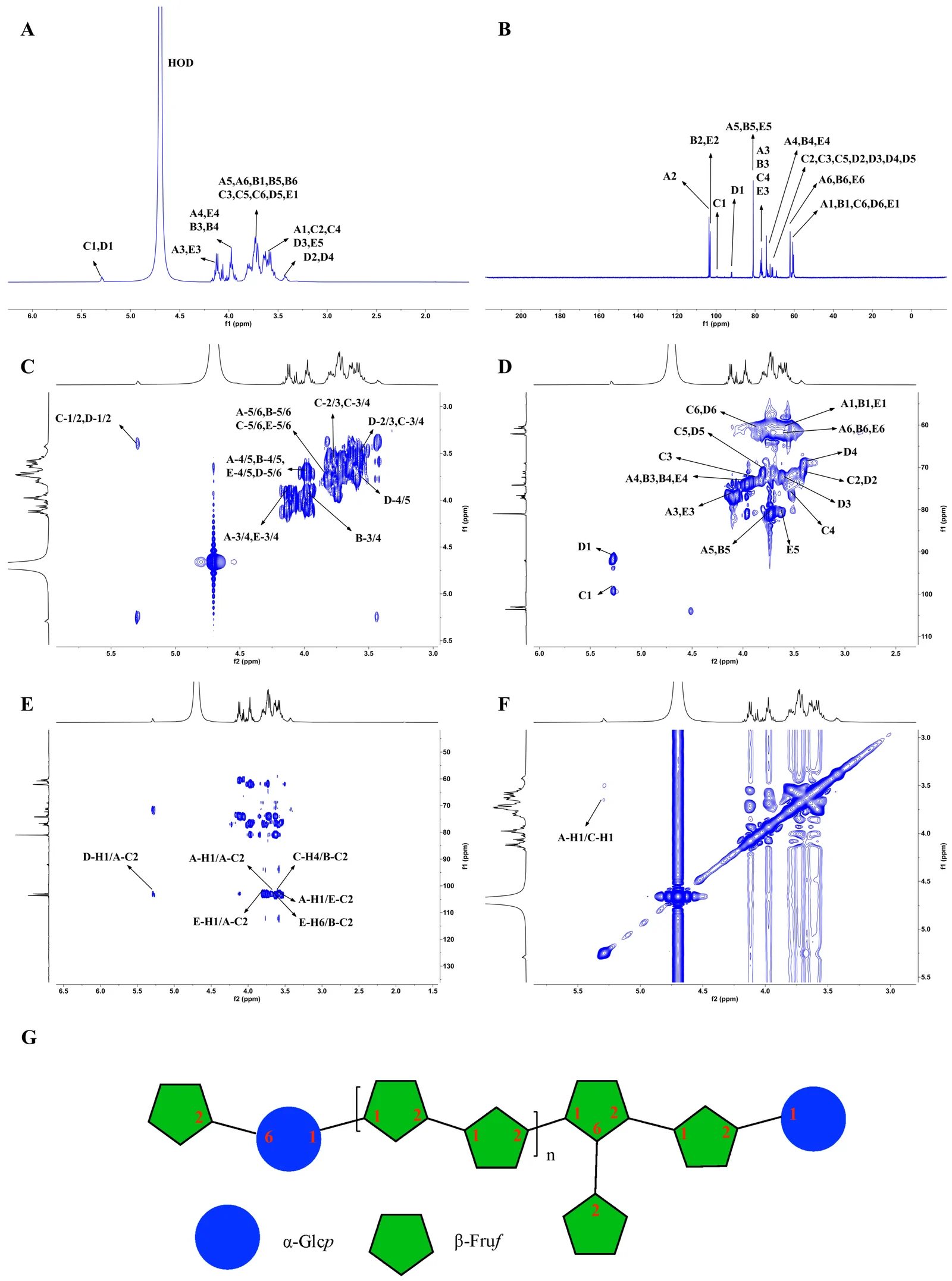
NMR spectra of AVP-1-2. (**A**) ^1^H spectrum; (**B**) ^13^C spectrum; (**C**) ^1^H–^1^H COSY spectrum; (**D**) HSQC spectrum; (**E**) HMBC spectrum; (**F**) NOESY spectrum; (**G**) Proposed representative schematic structural model of AVP-1-2.

In the ^1^H NMR (Figure 3A), no significant signals were observed around *δ* 4.4–5.2 ppm except for the HOD solvent peak (*δ* 4.71 ppm). This finding aligns with the presence of fructose. Two prominent signals at *δ* 5.3 ppm were attributed to α-configuration glucose residues (designated C and D). The *δ* 3.1–4.2 ppm region contained the majority of non-anomeric proton signals with severe overlap, which prompted further 2D NMR. The ^13^C NMR revealed five anomeric carbon signals at *δ* 103.61, 103.00, 99.47, 91.94, and 103.05 ppm (designated A–E) (Figure 3B). The signal at *δ*H/*δ*C 5.30/91.94 ppm (residue D) is consistent with a reducing-end α-D-glucopyranosyl residue.

The ^1^H and ^13^C chemical shifts in the main residues were assigned using COSY (Figure 3C) and HSQC (Figure 3D). In the anomeric region of the HSQC spectrum, cross-peaks for residues C and D were observed at *δ* 5.3/99.47 and *δ* 5.3/91.94 ppm, respectively. No anomeric cross-peaks were detected for residues A, B, and E, consistent with the structural characteristics of fructose [34]. The detailed results are shown in Table 4. Based on methylation analysis and literature comparison, residues A, B, and E were assigned as β-D-fructofuranosyl residues: →1)-β-D-Fruf-(2→ (residue A), β-D-Fruf-(2→ (residue B), and →1,6)-β-D-Fruf-(2→ (residue E). Residues C and D correspond to α-D-glucopyranosyl residues. The signal at *δ*H/*δ*C 5.30/91.94 ppm (residue D) is assigned to a reducing-end α-D-glucopyranosyl residue. Residue C is tentatively assigned as →6)-α-D-Glcp-(1→ based on the methylation data. The NMR data alone do not allow an unambiguous assignment of residue C because of its low abundance and spectral overlap [35,36,37,38].

The linkage was further elucidated by HMBC (Figure 3E) and NOESY (Figure 3F). The HMBC spectrum revealed key correlations, including A C2 (*δ* 103.61) to A H1 (*δ* 3.60), A C2 (*δ* 103.61) to D H1 (*δ* 5.30), A C2 (*δ* 103.61) to E H1 (*δ* 3.81), B C2 (*δ* 103.00) to C H4 (*δ* 3.55), B C2 (*δ* 103.00) to E H6 (*δ* 3.75), and E C2 (*δ* 103.05) to A H1 (*δ* 3.60). The NOESY spectrum showed a cross-peak between C H1 (*δ* 5.30) and A H1 (*δ* 3.60).

Collectively, the monosaccharide composition, methylation, and NMR data indicate that AVP-1-2 is an inulin-type fructan with a backbone composed mainly of →1)-β-D-Fruf-(2→. Glucose residues are minor components and are not part of the fructan backbone. The presence of minor →1,6)-β-D-Fruf-(2→ residues suggests limited branching, but the exact pattern and positions remain tentative. Figure 3G is therefore presented as a representative schematic model.

### 3.3. Anti-Inflammatory Activity of AVP-1-2 In Vitro

**Figure 4:**
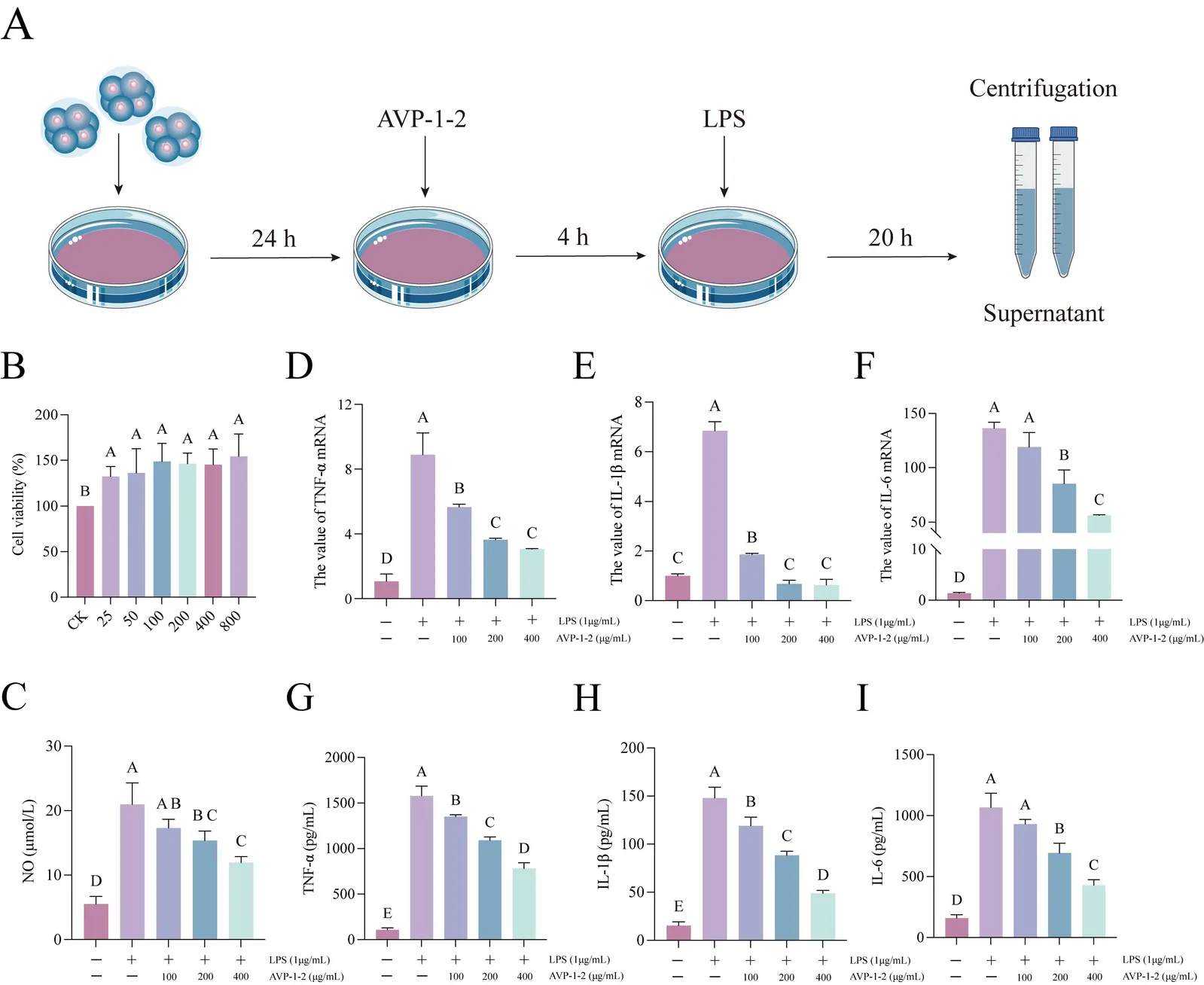
Anti-inflammatory activity of AVP-1-2 in RAW264.7 macrophages. (**A**) Cell experiment design; (**B**) Cell viability; (**C**) NO release; (**D**–**F**) mRNA levels and (**G**–**I**) Secreted levels of TNF-α, IL-1β, and IL-6. Values are expressed as mean ± SD (n = 3). Different letters indicate significant differences among groups at *p* < 0.05.

As shown in Figure 4A, the anti-inflammatory potential of AVP-1-2 was investigated in RAW264.7 macrophages. CCK-8 analysis revealed that AVP-1-2 exhibited no significant cytotoxicity at 25–800 μg/mL. No significant differences were observed among the different AVP-1-2 treatment concentrations (Figure 4B). Based on existing reports [28,39], concentrations of 100, 200, and 400 μg/mL were selected for the evaluation of anti-inflammatory activity in vitro. To further assess the inflammatory response, NO production was measured using the Griess assay (Figure 4C). LPS stimulation significantly increased NO production compared with the control group (*p* < 0.05). AVP-1-2 treatment reduced NO release in a concentration-dependent manner, reaching an inhibition rate of 43.08% at 400 μg/mL (*p* < 0.05). As illustrated in Figure 4D–F, TNF-α, IL-1β, and IL-6 mRNA showed significant increases (8.3-fold, 6.8-fold, and 99.5-fold) in LPS-stimulated cells compared with the control group. AVP-1-2 treatment lowered the mRNA abundance of these pro-inflammatory cytokines in a dose-dependent manner, with reductions of 65.54%, 90.79%, and 58.74% respectively at 400 μg/mL. The secreted levels of TNF-α, IL-1β, and IL-6 in the culture supernatant were further determined by ELISA. The secretion of these cytokines increased significantly after LPS stimulation (*p* < 0.05), whereas AVP-1-2 treatment lowered them in a concentration-dependent manner (Figure 4G–I). The inhibition rates of the 400 μg/mL group against these secreted levels were 50.53%, 66.91%, and 59.75%. These results indicate that AVP-1-2 could exert anti-inflammatory effects by suppressing pro-inflammatory cytokine expression and secretion, along with NO production.

## 4. Discussion

The anti-inflammatory activity of polysaccharides is closely linked to their structural characteristics, particularly molecular weight and monosaccharide composition [40]. For instance, low molecular weight fractions from *Torreya grandis* nuts [41], *Flammulina velutipes* [42], and *Lamiophlomis rotata* [43] have exhibited stronger anti-inflammatory effects than their high molecular weight counterparts. This phenomenon may be explained by the more efficient cellular uptake of low molecular weight polysaccharides [44], although this has not been directly confirmed. In the present study, the low molecular weight fructan AVP-1-2 (Mw 4.403 kDa) likewise exhibited notable anti-inflammatory activity. Further cell penetration studies may be considered to examine the cellular uptake of AVP-1-2. However, the relationship between molecular weight and anti-inflammatory activity is not strictly linear [45]. The high molecular weight fractions have also been reported to show strong anti-inflammatory activity in some studies, indicating that this association warrants further investigation [46,47,48]. With regard to monosaccharide composition, AVP-1-2 was found to consist of fructose and glucose. This compositional feature is similar to several known anti-inflammatory polysaccharides, including AMP from *Atractylodes macrocephala* [49], PMPS-A1 from *Pueraria montana* [50] and P-A from *Codonopsis pilosula* [51]. Collectively, the low molecular weight and the presence of fructose and glucose are considered key factors contributing to the anti-inflammatory activity of AVP-1-2.

In addition, AVP-1-2 exhibits typical features of inulin-type fructans [52]. The bioactivity of this type of fructan shows a degree of structural dependence [53]. The degree of polymerization (DP) is considered one of its key influencing parameters [54]. Studies have shown that long-chain (DP ≥ 23) inulin is superior to short-chain (DP ≤ 10) inulin in reducing inflammatory cytokine expression [55], alleviating liver lesions [56], and attenuating intestinal inflammation [57]. Beyond polymerization degree, branching structure also plays a key role in modulating anti-inflammatory activity. β-(2→6) branched fructans have been shown to attenuate pro-inflammatory responses by targeting Toll-like receptors [58]. Consistent with the structure–activity relationship discussed above, AVP-1-2 displayed an estimated degree of polymerization of approximately 27 and minor β-(2→6)-linked branching features. Therefore, the degree of polymerization and the branching structure are likely among the factors that affect the anti-inflammatory activity of AVP-1-2. It is worth noting that anti-inflammatory activity may not be determined by a single structural feature [53]. It has been reported that short-chain inulin exhibits enhanced anti-inflammatory effects in specific models, such as atherosclerosis [59]. This implies that the bioactivity of fructans likely results from the interplay of multiple structural parameters.

To determine whether AVP-1-2 shares common structural features with other APS, its structural features were compared with those reported for other *Allium* species. The β-(2→1)-linked fructosyl backbone with limited β-(2→6)-linked branches observed in AVP-1-2 is broadly consistent with fructans reported from *A. sativum* [60], *A. macrostemon* and *A. chinense* [61]. Its molecular weight of approximately 4.4 kDa is relatively low compared with those of many APS [10]. The corresponding DP was approximately 27, which falls within the intermediate range reported for *Allium* fructans. This structural information obtained for AVP-1-2 adds to the limited knowledge available for *A. victorialis* polysaccharides and further supports the reported prevalence of inulin-type fructans within this genus [11,62]. Given these similarities, it is plausible that AVP-1-2 exerts its anti-inflammatory effects through mechanisms similar to those proposed for other Allium polysaccharides, such as modulation of TLR4/NF-κB signaling [10]. Taken together, AVP-1-2 can be classified as an inulin-type fructan with structural characteristics typical of bioactive Allium polysaccharides.

## 5. Conclusions

In this study, a low molecular weight inulin-type fructan (AVP-1-2, 4.403 kDa) was isolated from *A. victorialis*. Structural characterization revealed that AVP-1-2 is composed of fructose and glucose, with a backbone consisting of →1)-β-D-Fruf-(2→ and minor →1,6)-β-D-Fruf-(2→ branches. This structural type is commonly reported in Allium polysaccharides. In LPS-stimulated RAW264.7 macrophages, AVP-1-2 dose-dependently suppressed LPS-induced inflammatory markers (TNF-α, IL-1β, IL-6, and NO), thereby exerting in vitro anti-inflammatory effects. These findings support the potential of AVP-1-2 as a bioactive fructan and provide a structural basis for the development of functional foods derived from *A. victorialis*. However, the molecular mechanisms underlying its activity remain unclear, and future studies should focus on validating the in vivo efficacy of AVP-1-2 and elucidating the relevant signaling pathways.

## Figures and Tables

**Table 1:** Monosaccharide composition of AVP-1-2.

Monosaccharide Composition	Molar Ratio (%)
Fructose	89.66%
Glucose	10.34%

**Table 2:** Molecular weight analysis of AVP-1-2. Mn, number-average molecular weight; Mp, peak molecular weight; Mw, weight-average molecular weight; Mz, z-average molecular weight.

Mn (kDa)	Mp (kDa)	Mw (kDa)	Mz (kDa)	Polydispersity (Mw/Mn)
4.316	3.784	4.403	4.518	1.02

**Table 3:** Glycosidic bonding patterns in AVP-1-2 based on methylation analysis.

Linkage Type	PMAAs	RT	Mass Fragments	Mw	Molar Ratio
Fruf-(2→	2,5-di-O-acetyl-(2-deuterio)-1,3,4,6-tetra-O-methyl hexitols (mannitol,glucitol)	7.995	87, 101, 102, 129, 161, 162, 205	323	17.21
Glcp-(1→	1,5-di-O-acetyl-2,3,4,6-tetra-O-methyl glucitol	9.528	87, 102, 118, 129, 145, 161, 162, 205	323	1.75
→1)-Fruf-(2→	1,2,5-tri-O-acetyl-(2-deuterio)-3,4,6-tri-O-methyl hexitols	13.087	87, 101, 129, 145, 161, 190	351	24.32
→1)-Fruf-(2→	1,2,5-tri-O-acetyl-(2-deuterio)-3,4,6-tri-O-methyl hexitols	13.209	87, 101, 129, 145, 161, 190	351	46.16
→6)-Glcp-(1→	1,5,6-tri-O-acetyl-2,3,4-tri-O-methyl glucitol	14.462	87, 99, 102, 118, 129, 162, 189, 233	351	7.87
→1,6)-Fruf-(2→	1,2,5,6-tetra-O-acetyl-(2-deuterio)-3,4-di-O-methyl hexitols	18.999	87, 99, 100, 129, 189, 190	379	1.21
→1,6)-Fruf-(2→	1,2,5,6-tetra-O-acetyl-(2-deuterio)-3,4-di-O-methyl hexitols	19.197	87, 99, 100, 129, 189, 190	379	1.47

The two peaks assigned to →1)-Fruf-(2→ and the two peaks assigned to →1,6)-Fruf-(2→ represent epimeric alditol derivatives formed during NaBD_4_ reduction of the same linkage type.

**Table 4:** ^1^H and ^13^C NMR chemical shift assignments (ppm) for AVP-1-2.

Label	Glycosyl Residues	H1/C1	H2/C2	H3/C3	H4/C4	H5/C5	H6/C6
A	→1)-β-D-Fruf-(2→	3.60/60.79	—/103.61	4.14/77.29	3.99/74.17	3.74/80.98	3.72, 3.63/62.08
B	β-D-Fruf-(2→	3.81/61.19	—/103.00	4.07/76.65	4.04/74.24	3.77/81.20	3.67, 3.59/62.17
C	→6)-α-D-Glcp-(1→	5.30/99.47	3.52/71.51	3.86/73.68	3.55/76.88	3.73/71.38	3.71/60.69
D	α-D-Glcp-(1→	5.30/91.94	3.44/71.01	3.65/72.35	3.44/69.02	3.82/71.44	3.69/60.06
E	→1,6)-β-D-Fruf-(2→	3.81/60.35	—/103.05	4.15/77.17	4.00/74.24	3.66/81.08	3.75/62.28

Residue C is tentatively assigned as →6)-α-D-Glcp-(1→ based on methylation analysis. The NMR data alone do not allow an unambiguous assignment.

## Data Availability

The raw data supporting the conclusions of this article will be made available by the authors on request.

## Supplementary Materials

The following supporting information can be downloaded at https://www.mdpi.com/article/10.3390/plants15172687/s1. Table S1: Gradient elution program for monosaccharide analysis. Table S2: Concentration gradients of mixed monosaccharide standards. Table S3: Calibration curves for monosaccharide standards. Table S4: Fluorescence quantitative primers. Table S5: Factor and levels of extraction process. Table S6: Orthogonal experiment results. Figure S1: Photographs of *A. victorialis* during field collection. Figure S2: Molecular configuration analysis diagram of AVP-1-2. Figure S3: Scanning electron microscopy (SEM) images of AVP-1-2: (A) 5000×; (B) 10,000×. Figure S4: Total ion chromatogram of AVP-1-2. Figure S5: EI mass spectra of the PMAA derivatives identified from AVP-1-2. (A) Fruf-(2→; (B) Glcp-(1→; (C) →1)-Fruf-(2→ (peak 1); (D) →1)-Fruf-(2→ (peak 2); (E) →6)-Glcp-(1→; (F) →1,6)-Fruf-(2→ (peak 1); (G) →1,6)-Fruf-(2→ (peak 2).
