# Supplementary material for: Structural Characterization and Anti-Inflammatory Effects in LPS-Stimulated RAW264.7 Macrophages of an Inulin-Type Fructan (AVP-1-2) Isolated from Allium victorialis L
Source: Plants (Basel). 2026 Sep 1;15(17):2687. doi: 10.3390/plants15172687 (PMC13567278; doi:10.3390/plants15172687)
Supplement: Supplementary file 1 [file plants-15-02687-s001.zip › plants-4493866-supplementary.pdf]

Table S1 Gradient elution program for monosaccharide analysis..

Time (min)	A (ddH2O)	B (0.1 M NaOH)	C (0.1 M NaOH–0.2 M NaAc)
0	95	5	0
26	85	5	10
42	85	5	10
42.1	60	0	40
52	60	40	0
52.1	95	5	0
60	95	5	0

Table S2 Concentration gradients of mixed monosaccharide standards.

Monosaccharide	Concentration (μg/mL)							
Fuc	0.4	0.8	4	8	16	24	32	40
Rha	0.4	0.8	4	8	16	24	32	40
Ara	0.4	0.8	4	8	16	24	32	40
Gal	0.4	0.8	4	8	16	24	32	40
Glc	0.4	0.8	4	8	16	24	32	40
Xyl	0.4	0.8	4	8	16	24	32	40
Man	0.5	1	5	10	20	30	40	50
Fru	0.6	1.2	6	12	24	36	48	60
Rib	0.6	1.2	6	12	24	36	48	60
Gal-UA	0.6	1.2	6	12	24	36	48	60
Gul-UA	0.6	1.2	6	12	24	36	48	60
Glc-UA	0.6	1.2	6	12	24	36	48	60
Man-UA	0.6	1.2	6	12	24	36	48	60

Table S3 Calibration curves for monosaccharide standards.

Monosaccharide	Retention time (min)	Slope	Correlation coefficient (R^2)
Fuc	4.3668	0.9105	0.9976
Rha	8.6418	0.9991	0.9912
Ara	9.5835	1.0344	0.9982
Gal	12.3335	1.1235	0.9992
Glc	14.0668	1.1847	0.9992
Xyl	16.7585	1.095	0.9965
Man	17.2918	0.9163	0.9979
Fru	20.3835	0.2954	0.9998
Rib	22.9585	1.0046	0.9996
Gal-UA	34.6335	0.2994	0.9986
Gul-UA	35.4418	0.3704	0.9985
Glc-UA	37.1585	0.5238	0.9994
Man-UA	39.0252	0.3121	0.9991

Table S4 Fluorescence quantitative primers.

Gene	Primer name	Primer sequence
TNF-α	TNF-α-F	5’-GCCACCACGCTCTTCTGTCTACT-3’
	TNF-α-R	5’-TGGTTTGTGAGTGTGAGGGTCTGG-3’
IL-1β	IL-1β-F	5’-ATCTCGCAGCAGCACATCAACAAG-3’
	IL-1β-R	5’-GGTCCACGGGAAAGACACAGGTAG-3’
IL-6	IL-6-F	5’-CCTTCTTGGGACTGATGCTGGTGA-3’
	IL-6-R	5’-AAGCCTCCGACTTGTGAAGTGGTAT-3’

Table S5 Factor and levels of extraction process.

Levels	Factor		
	A/(g/mL)	B/h	C/℃
1	1:10	2	85
2	1:15	3	90
3	1:20	4	95

Table S6 Orthogonal experiment results.

Number	A/(g/mL)	B/°C	C/h	Yield（%）
1	1	1	1	1.772
2	1	2	2	1.369
3	1	3	3	1.341
4	2	1	2	1.507
5	2	2	3	1.506
6	2	3	1	1.134
7	3	1	3	0.702
8	3	2	1	0.952
9	3	3	2	0.753
K1	1.494	1.327	1.286	
K2	1.382	1.276	1.210	
K3	0803	1.076	1.183	
R	0.691	0.251	0.103	

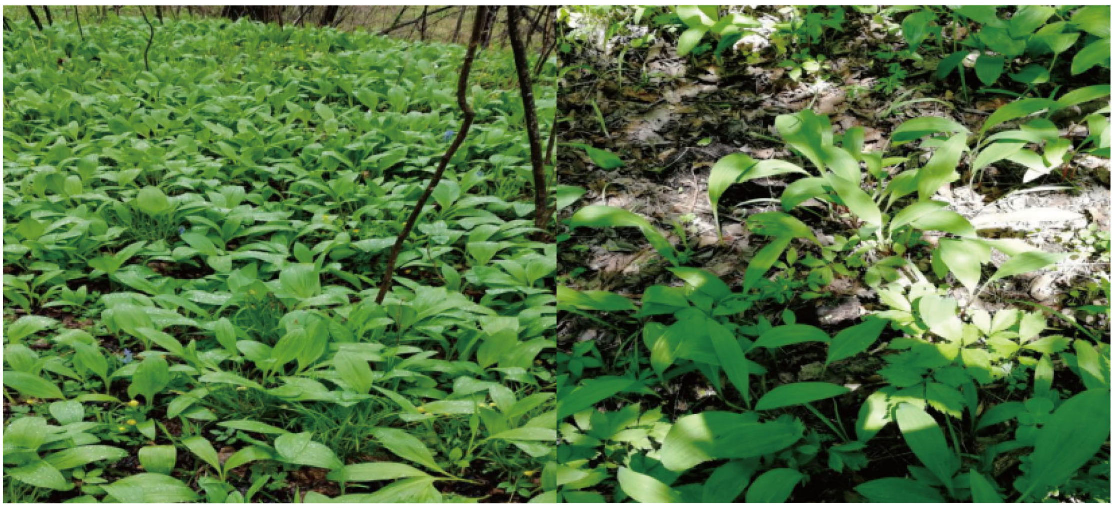

Figure S1 Photographs of *A. victorialis* during field collection.

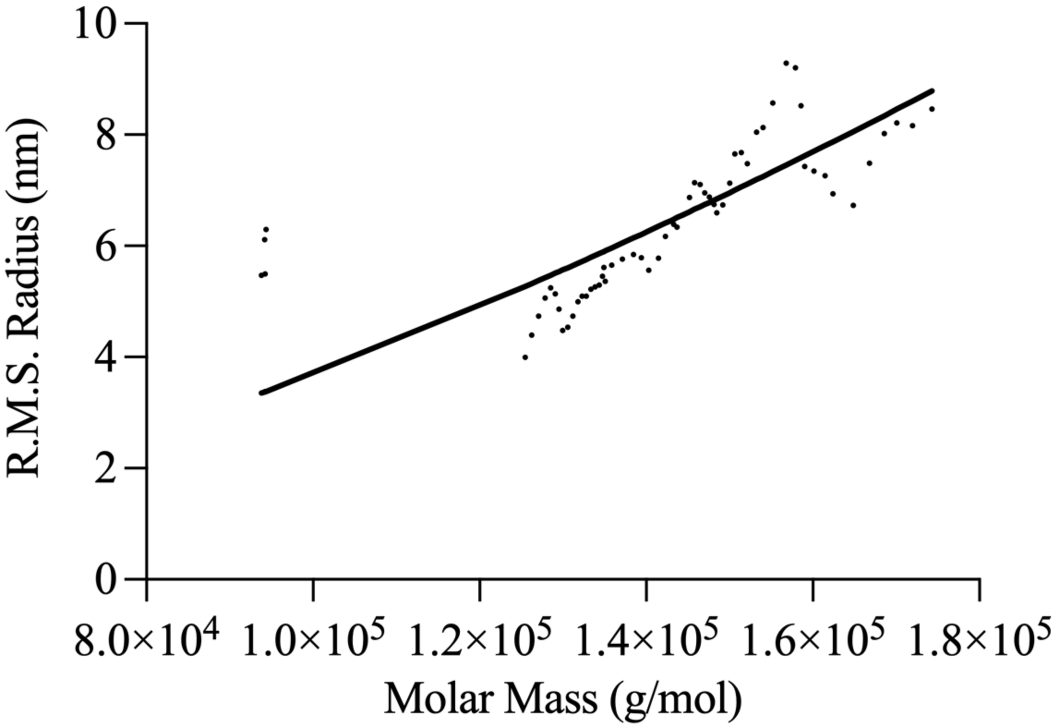

Figure S2 Molecular configuration analysis diagram of AVP-1-2.

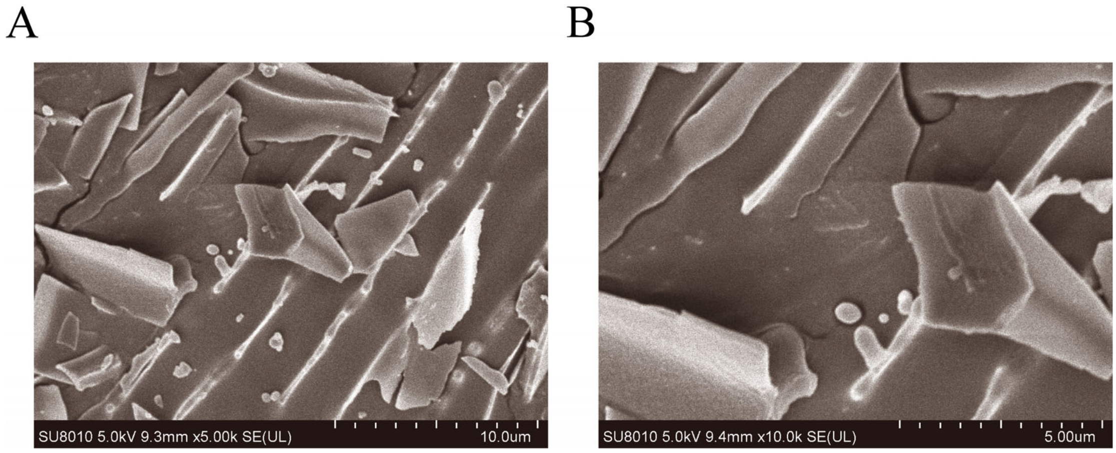

Figure S3 Scanning electron microscopy (SEM) images of AVP-1-2: (A) 5000×; (B) 10000×.

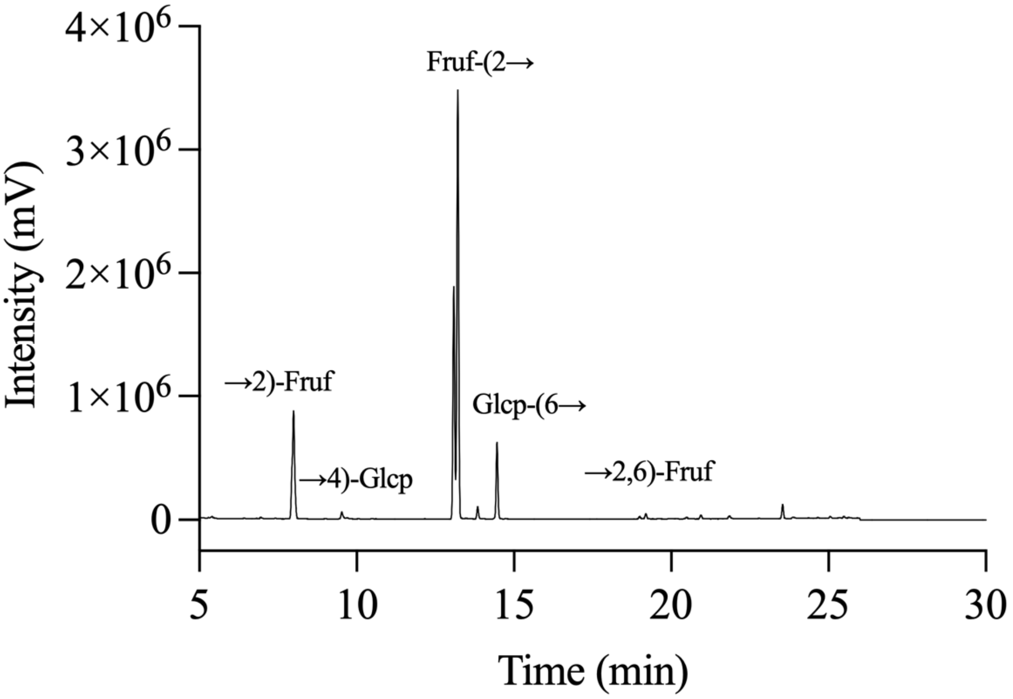

Figure S4 Total ion chromatogram of AVP-1-2.

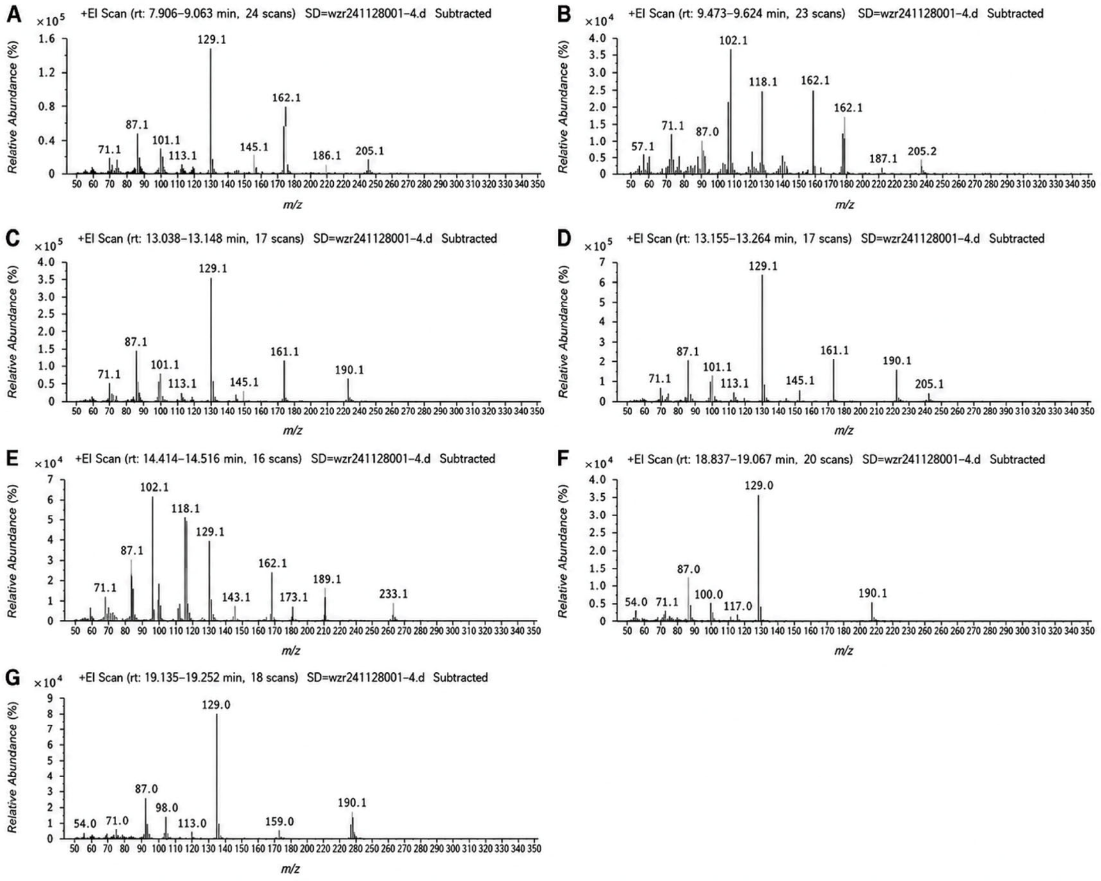

Figure S5 EI mass spectra of the PMAA derivatives identified from AVP-1-2. (A) Fruf-(2→; (B) Glcp-(1→; (C) →1)-Fruf-(2→ (peak 1); (D) →1)-Fruf-(2→ (peak 2); (E) →6)-Glcp-(1→; (F) →1,6)-Fruf-(2→ (peak 1); (G) →1,6)-Fruf-(2→ (peak 2).
